# Interpretation of detections of volcanic activity at Ioto Island obtained from *in situ* seismometers and remote hydrophones of the International Monitoring System

**DOI:** 10.1038/s41598-019-55918-w

**Published:** 2019-12-20

**Authors:** Hiroyuki Matsumoto, Mario Zampolli, Georgios Haralabus, Jerry Stanley, James Mattila, Nurcan Meral Özel

**Affiliations:** 1 Japan Agency for Marine-Earth Science and Technology (JAMSTEC), 2-15, Natsushima, Yokosuka 237-0061 Japan; 2 Comprehensive Nuclear-Test-Ban Treaty Organization (CTBTO), Vienna International Centre, P. O. Box 1200, 1400 Vienna, Austria

**Keywords:** Natural hazards, Volcanology

## Abstract

*In-situ* seismic observations identified that volcanic activity of Ioto (formerly Iwojima), a volcanic island offshore Japan, increased in early September 2018. Observations of discolored nearshore waters and a splash reported by a local flyover provided evidence for a connection between undersea eruptions and recorded seismic activity. However there remain uncertainties as to when the undersea eruption series commenced and how much of the *in-situ* seismic activity recorded on the island was associated with volcanic earthquakes versus undersea eruptions. During this period, a large number of underwater acoustic (hydroacoustic) signals were recorded by the Comprehensive Nuclear-Test-Ban Treaty (CTBT) International Monitoring System (IMS) hydroacoustic station HA11, at Wake Island (U.S. Territory), in the northwestern Pacific Ocean with signals with directions of arrival consistent with sources located at Ioto. The analysis presented here interprets signal features of the remote hydroacoustic recordings provided by HA11 in order to attempt to distinguish between volcanic earthquake signals and undersea eruption signals originating from Ioto. Histograms of hydroacoustic events interpreted as originating from Ioto correlate well with the *in-situ* seismic observations at Ioto in the early stage of volcanic activity. The results presented suggest that around 75% of the signals detected at HA11 with directions of arrival consistent with Ioto as their origin could be associated with undersea eruptions, supporting the conclusion that the IMS hydroacoustic stations can contribute to volcanic event remote monitoring.

## Introduction

Substantial volcanic activity occurs along the Izu-Ogasawara-Mariana arc volcanic chain of islands and seamounts where the Pacific plate is subducting beneath the overlaying Philippine Sea plate^1,2^. Whilst monitoring of volcanic activity on land can be conducted using *in-situ* geophysical, geochemical, and satellite based radar technologies^3^, the monitoring of submarine volcanoes is more problematic as it requires either numerous local observations, e.g. *in-situ* visual, seismic and/or acoustic observations of undersea volcanic activity, or the application of remote techniques such as underwater acoustic (hydroacoustic) detections coupled with event discrimination. This paper addresses the subject of monitoring submarine volcanic activity with such remote hydroacoustic techniques, using data recorded by hydrophones of the Comprehensive Nuclear-Test-Ban Treaty (CTBT) International Monitoring System.

Hydrophones have previously shown their effectiveness in detecting hydroacoustic signals associated with undersea volcanic activity over long distances. In the 1950s, it was demonstrated that hydroacoustic events originating from the Myojin volcano in Japan were detected on hydrophone arrays deployed within the Sound Fixing And Ranging (SOFAR) channel axis at a distance of approximately 8,000 km^4^. The SOFAR channel acts as a natural acoustic waveguide for hydroacoustic waves and minimizes energy loss from interaction with boundaries, thus enabling the detection of events over long ranges^5–7^. Hydroacoustic signals from high intensity acoustic events can be detected at even greater ranges, for example hydroacoustic signals associated with the Monowai undersea eruptions in 2011 and the Ahyi undersea eruptions in 2014 were detected by hydrophones at a distance in excess of 15,000 km^8,9^.

During the 1990s and 2000s, a number of experiments with *in-situ* and remote hydroacoustic monitoring sensors were conducted to study undersea volcanic activity^10–14^. For example, ocean bottom hydrophone observations were employed for the *in-situ* measurements of undersea volcanic activity of the Vailulu’u seamount in the Territory of American Samoa and of the Brothers volcano in New Zealand in the southwestern Pacific Ocean, these observations contributing to studies of the local seismicity and the internal dynamics associated with magma injection processes of submarine volcanoes^15,16^.

A study conducted in 2006 consisting of direct visual observations^17^ and *in-situ* acoustic measurements performed by a Remotely Operated Vehicle (ROV) equipped with a hydrophone^18^ investigated undersea volcanic eruptions from the Northwest Rota-1 seamount of the Northern Mariana Islands. Moreover, a hydrophone moored at Northwest Rota-1 seamount during the period 2008–2009, along with other turbidity and temperature sensors, were used to investigate the gas flux from explosive events^19^ and to detect a volcanic-eruption-landslide sequence during another 1-year deployment in 2009–2010^20^. On-site investigation and visual observations of an active boninite eruption occurring at the West Mata submarine volcano at a depth of 1,200 m in the northeast Lau Basin in the southwestern Pacific Ocean, with recordings from an *in-situ* hydrophone were used to characterize the frequency content of magma bubble bursts and eruptive degassing^21,22^.

The hydroacoustic signals associated with submarine eruptions at Axial seamount, on the Juan de Fuca Ridge in the northeastern Pacific Ocean were detected by remote water-column hydrophone arrays^23,24^. More recently the 2015 undersea eruption of Axial seamount was monitored using an ocean bottom seismometer array together with hydrophones of the Ocean Observatory Initiative (OOI) Cabled Array^25,26^. During the undersea eruption, the recorded hydroacoustic signals were used to investigate the eruption mechanism^27^.

On-land seismic sensors can detect seismic signals originating from hydroacoustic signals, for example the Polynesian seismic network was used to detect such signals associated with volcanic activities in the Tahiti-Mehetia area, enabling the discrimination between volcanic events and earthquake activity^28,29^. Additionally, ocean bottom seismometers were used to record seismo-acoustic arrivals from undersea volcanoes in the northwestern Pacific Ocean^30,31^; and a combination of a seismic network on a volcanic island and water-column hydrophones were used to identify seismic events originating from the vicinity of the Cape Verde Island in the Atlantic Ocean^32^. A combination of a regional seismic network and distant water-column hydrophones was also used to study the 2014 undersea eruption of Ahyi seamount in the northwestern Pacific Ocean^9^.

On a global scale, the establishment and certification of hydroacoustic (HA) stations of the CTBT International Monitoring System (IMS) have progressed continuously since the year 2000. These hydrophone triplet stations monitor continuously the world’s oceans for signs of nuclear explosions^33,34^. The IMS HA network comprises six hydrophone stations and five T-phase stations. T-phase stations utilize on-shore broadband seismometers to detect seismic arrivals generated by hydroacoustic waves which couple into seismic waves near the shore.

**Figure 1:**
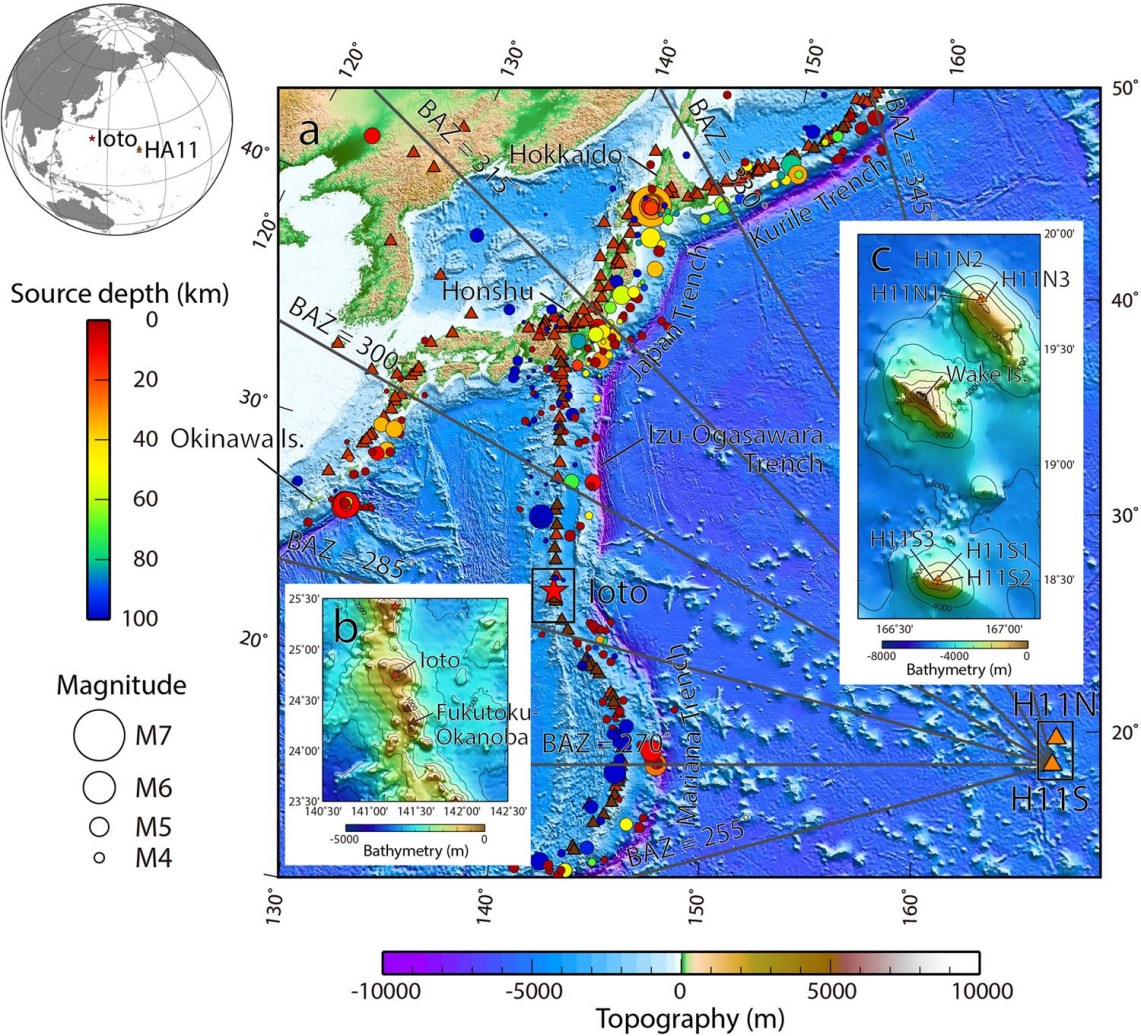
The northwestern Pacific Ocean area covered by the study, showing (**a**) the locations of the Ioto (formerly Iwojima) volcanic island (red star) and the IMS HA11 hydroacoustic station at Wake Island (orange triangles). The distance between Ioto and HA11 is about 2,700 km. HA11 comprises two triplets of hydrophones, referred to as H11N and H11S. Circles mark the locations of the 6,165 earthquakes recorded during the month of September 2018, their color and size represent source depth and magnitude, respectively. Dark and bright red triangles locate above-surface and submarine volcanoes, respectively, as registered by the Smithsonian Global Volcanism Program. Radial marks are back-azimuths (BAZ) from H11S. (**b**) Detailed map of Ioto and its surroundings. Ioto is categorized as an above sea-level volcano and is one of the volcanic islands in the Izu-Ogasawara-Mariana arc volcanic chain. Fukutoku-Okanoba is located 55 km south from Ioto, and is categorized as a submarine volcano. (**c**) Detailed map of the Wake Island area, with the North and South triplet hydrophones labeled H11N1 through H11N3 and H11S1 through H11S3, respectively. Figures were generated with the GMT software^54^, using the GEBCO 30-s in arc bathymetry dataset^55^ and the earthquake event catalogue issued by the International Seismological Centre (ISC)^56^.

The hydrophone stations utilize hydrophones which are suspended in the water-column by vertical moorings in the SOFAR channel to ensure optimal acoustic coverage. Each mooring consists of an anchor, a riser cable, a hydrophone and a submerged float. Each station is composed of two triplets of closely spaced hydrophones, one located off-shore to the north and the other one located off-shore to the south of an island, except for HA01 Cape Leeuwin (Australia) which has only one triplet. Each triplet is configured as an approximately equilateral triangle with sides of 2 km, with a hydrophone mooring located at each corner of the triangle. Signals are recorded with a sampling frequency of 250 Hz, and the signal bandwidth is 100 Hz. The digital raw data recorded by the hydrophones are sent in near-real time to the CTBT’s International Data Centre (IDC) in Vienna, Austria, using a dedicated satellite link. The CTBT IMS HA station HA11, at Wake Island, used in this study, is located in the northwestern Pacific Ocean (Fig. 1). It comprises one triplet located 54 km to the North (H11N) and another one located 89 km to the South (H11S) of Wake Island (Fig. 1c). The hydrophones are deployed at a water depth of approximately 750 m^35^.

Ioto (formerly known as Iwojima) is a volcanic island located at the boundary between the Izu-Ogasawara Island and the Mariana Island volcanic chains, which parallel the undersea trenches having the same names (Fig. 1a). Ioto Island is formed by the top of a caldera approximately 40 km wide, rising 2 km above the sea-floor (Fig. 1b). The highest elevation of the island is 161 m above sea-level. Documented volcanic activity of Ioto dates back to 1889 when a phreatic eruption occurred, creating a 50 m diameter crater^36^. It has been reported that phreatic eruptions, mud discharge or mud overflows occurred repeatedly on the island at intervals ranging between a few years to tens of years. Additionally, discolored waters have been frequently observed in the nearshore area, providing evidence of undersea eruptions. The intense activity of Ioto warranted the installation of volcano monitoring equipment by the Japan Meteorological Agency (JMA) and other governmental institutions, including seismometers, video, Global Positioning System (GPS) stations, and infrasound sensors. All these *in-situ* observation platforms are currently operational. The JMA issues Monthly Reports of volcanic activity in Japan, in which the number of volcanic earthquakes per day is reported to the public. These reports show that volcanic seismicity at Ioto increased during the month of September 2018^37^. It should be noted that this study did not investigate the amplitude of the *in-situ* seismic observations or the hypocenters of the volcanic earthquakes, as the only data that were available to the authors were the hourly detections from the *in-situ* seismometer. Therefore, seismic event counts at Ioto were compared with the remote hydrophone data, as the authors did not have access to the raw seismic data. Local flyover observations conducted by the Japan Maritime Self-Defense Force (JMSDF) observed, on one occasion, discolored water and a water splash of up to 10 m in the nearshore shallow-water area during this time (Supplementary Fig. 1). These visual signs provided evidence of a connection between undersea eruptions and recorded seismic activity. However, it is generally not possible to distinguish undersea eruptions from seismic activity using only the counts of earthquakes obtained from the *in-situ* seismometer recordings.

Coincident with the Ioto volcanic activity in September 2018, a large number of hydroacoustic signals were recorded by the HA11 hydrophone triplets located 2,700 km southeast of Ioto. In the study presented here, hydroacoustic signals recorded by HA11 are examined during this period with the purpose of identifying events originating from Ioto and comparing these with the *in-situ* seismic observations. Further analysis attempted to categorize the detected signals into either volcanic earthquakes or undersea eruptions based on the signal duration and the frequency content.

## Results

### Characteristics of hydroacoustic signals originating from Ioto

According to the measured *in-situ* seismicity, Ioto exhibited increased activity starting around 17:00 UTC on 07 September 2018. This activity decreased gradually, except for a spike on 12 September 2018, until it returned to levels comparable with the usual background activity around 21 September 2018. Our examination of the hydroacoustic signals at HA11 during the entire period of September 2018 suggests that signals associated with activity from Ioto were occurring as early as 03 September 2018, as discussed below.

**Figure 2:**
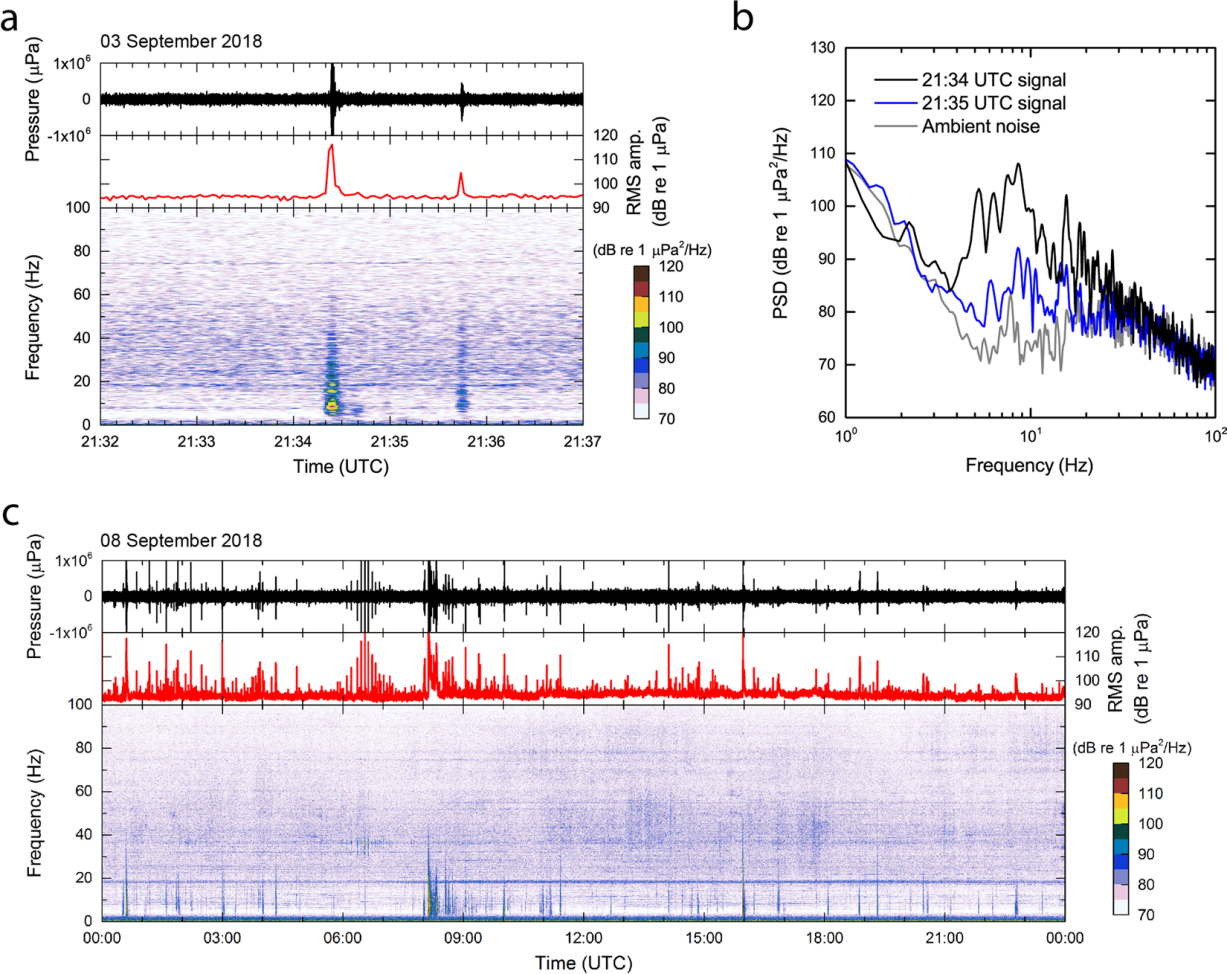
H11S1 hydroacoustic data associated with interpreted undersea eruptions. The waveforms have been corrected with the frequency-amplitude-phase (FAP) response of the hydrophone (Supplementary Fig. 2b) and filtered with a 6–60 Hz pass-band before displaying the time-series. (**a**) An example of a typical recording associated with the Ioto volcanic activity on 03 September 2018 with 2 events (i.e. centered at 21:34:24 UTC and 21:35:44 UTC). The root-mean-square (RMS) amplitude, using 2-s bins, is also shown. (**b**) Hydroacoustic power spectral densities (PSDs) of the signals received over the time period of 21:34:20 UTC to 21:34:40 UTC, 21:35:40 UTC to 21:36:00 UTC, and ambient noise. (**c**) Recordings showing arrivals from the volcanic activity associated with Ioto on 08 September 2018, presented in a format similar to (**a**).

An example of a waveform recorded by hydrophone H11S1 (the first hydrophone of the H11S triplet) is presented in Fig. 2a (after removal of direct current [DC] offset, correction with the Frequency-Amplitude-Phase [FAP] response of the hydrophone [the raw time-series and the FAP response of the hydrophone are shown in Supplementary Fig. 2], and pass-band filtering between 6 Hz and 60 Hz using a second order Butterworth filter), including also the root-mean-square (RMS) amplitude and the spectrogram for a time-window following the onset of hydroacoustic events on 03 September 2018. The short duration signals recorded at 21:34:24 UTC and 21:35:44 UTC were both associated with the Ioto volcanic activity based on their direction of arrival. The RMS amplitude and frequency band of these events differ between the arrivals (Fig. 2a). However, our examination of the HA11 recordings from this period suggests that the Ioto volcanic activity events can be characterized by a short duration, typically less than 20-s, which can be distinguished from that of harmonic tremors and earthquakes, which generally last longer than 20-s.

Power spectral densities (PSDs) of the above signals received at H11S1 from Ioto at 21:34:24 UTC and 21:35:44 UTC computed using a 20-s time-window, and ambient noise, obtained from a 20-s period during which there were no identified signal arrivals, are compared in Fig. 2b. The ambient noise PSD is consistent with the typical ocean background noise known from the literature^38,39^. The PSD of the 21:34:24 UTC signal exceeds the background noise by 20–25 dB in the band between 3 Hz and 30 Hz.

Another example of an H11S1 hydrophone waveform, RMS amplitude, and spectrogram is shown in Fig. 2c. This example is from the relatively high level of volcanic activity recorded during the entire day of 08 September 2018. The figure was created with the same processing used to generate Fig. 2a. Signals identified between 00:00 UTC and 04:00 UTC are primarily associated with the Ioto volcanic activity. A longer duration low-frequency arrival recorded around 08:10 UTC is not associated with Ioto as it was from a back-azimuth of 260°, a direction which differs substantially from the back-azimuth to Ioto.

Back-azimuths of the discrete signals identified between 06:00 UTC and 07:00 UTC vary with time and are not consistent with signals originating at Ioto. Previous studies of long-term ambient noise have shown significant whale vocalizations in the 15–25 Hz band in signals recorded on IMS hydrophones^40^. Based on similar previous observations^41–43^ the occurrence of a nearly continuous ambient noise train around 18 Hz (Fig. 2c) is thus attributed to whale vocalizations. At frequencies above the whale vocalizations the signals of interest associated with Ioto can be distinguished from the background noise up to ~60 Hz.

### Direction-of-arrival and number of hydroacoustic signals associated with Ioto volcanic activity

Cross-correlation analysis^44,45^ of the seismo-acoustic signals received by the HA11 hydrophone triplets during September 2018 was used to determine the Direction-of-Arrival (DOA) to help interpret their origin. The DOA was evaluated by using the cross-correlation function between two hydrophones’ waveforms by a method analogous to the one presented in reference^44^, equations (1) and (2).

The following parameters were used in the cross-correlation processing:

1. The time-window was set to 20-s in order to enable the detection of the impulsive signals characterizing the Ioto volcanic activity. Substantially longer duration signals arriving from the same direction are interpreted to be from seismic activity. Using a time window shorter than 20-s was found to lead to an over-estimation of the number of arrivals.
2. A time-window overlap of 5-s was found to be optimal for this analysis; no overlap resulted in no detection of arrivals spanning two adjacent time-windows, whereas an overlap substantially larger than 5-s was found to lead to double counting of the same arrival.
3. A second-order band-pass Butterworth filter was applied in different bands to the signals before cross-correlation. Examining the data in different frequency bands enabled distinguishing between signals interpreted to be undersea eruptions and those interpreted to be volcanic earthquakes. Further details regarding the filters chosen in this context are provided in the Discussion section.

Discrimination between T-phases (earthquake generated waves which have originated in the crust and successively coupled into the water-column, reaching the hydrophones as hydroacoustic waves) and direct seismic P-waves (seismic waves travelling through the crust and converted to acoustic signals at the sea-floor near the hydrophone triplet) was achieved by filtering apparent horizontal velocity. This apparent velocity was determined by the inter-hydrophone arrival delay at the hydrophone triplet; P-wave crustal velocities are high, typically 5 to 8 km/s near the Earth surface^46^, and usually their apparent horizontal velocity exceeds these speeds because the waves couple from the sediment to the water near the hydrophone with a steep angle. The velocities of T-phases and H-phases (H-phase refers to a signal which originated from a source in the water and propagated through the water-column to a hydrophone) are typically 1.48 km/s ± 10%.

To discriminate between arrivals considered as valid, and arrivals considered non-physical (i.e. not associated with a propagating wave reaching the hydrophone triplet), we used the residual, defined as the sum of the delay between arrivals at each pair of the three hydrophones. For valid arrivals this residual should ideally be zero, however due to positional uncertainties of the hydrophone’s mooring base, and the actual location of the hydrophones, which are subjected to motions induced by water currents, non-zero residuals can be expected. In this study, the residual threshold for rejecting DOA estimations was set to 0.15-s which is based on realistic hydrophone positional uncertainties.

**Figure 3:**
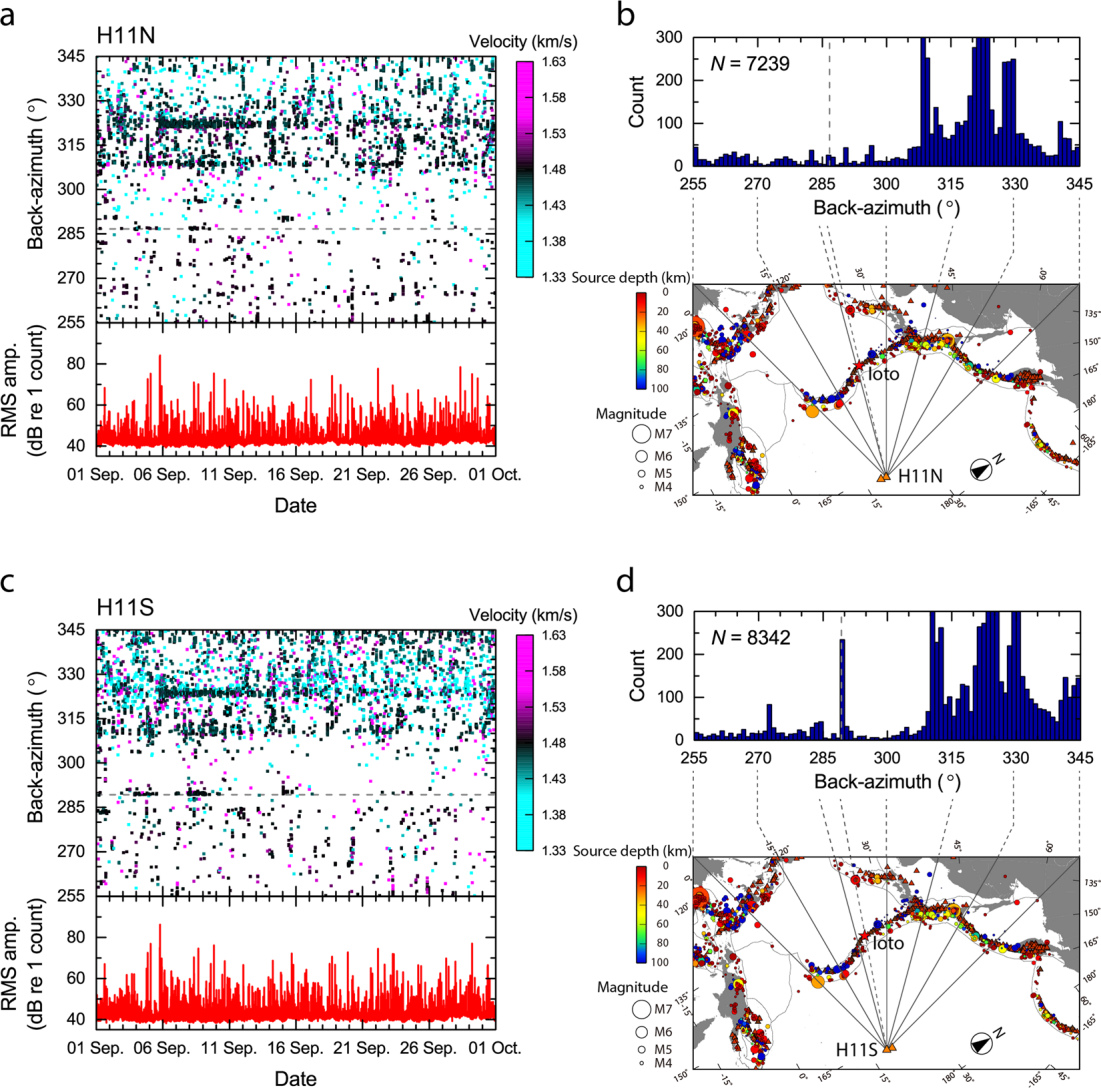
Cross-correlation analysis of data with a band-pass filter between 6 Hz and 60 Hz during the entire month of September 2018. (**a**) Back-azimuth between 255° and 345° of the signals detected by H11N together with the RMS amplitude of the band-pass filtered signal. Each dot is calculated over a 20-s time window. Dashed line represents the true back-azimuth to Ioto. (**b**) Histogram of event count *vs*. back-azimuth and map of the northwestern Pacific Ocean with lines of back-azimuth from H11N at Wake Island (note north is not up). Figures (**c**) and (**d**) are the analogous results for H11S. The numbers of hydroacoustic signal detections from Ioto identified by H11N and H11S differ, probably due to differences in the bathymetry along the two travel paths. The back-azimuths suggest that the Ioto volcanic activity had two separate periods of high activity between 03 and 10 September with a 2-day gap. Hydroacoustic signals associated with the 05 September earthquake of Hokkaido (back azimuth 324.3° to the epicenter) and its aftershocks were also clearly detected at H11S. These seismic source arrivals are found until the end of the analysis period, although they decrease in number with time.

The results of the cross-correlation analysis for the entire month of September 2018 are presented in Fig. 3a,b for the North triplet (H11N) and in Fig. 3c,d for the South triplet (H11S). In Fig. 3a,c, each point represents the back-azimuth of one or more hydroacoustic signals detected in each time-window by the cross-correlation analysis. The time resolution of each point is 15-s, resulting from the combination of time-window and overlap. These back-azimuth plots, zoomed in around the DOA associated with Ioto, namely 286.7° from H11N and 289.3° from H11S, between 01 and 16 September 2018 are shown in Supplementary Fig. 3. High seismicity associated with the Japan Trench, from the same general area where the large Tohoku earthquake occurred in March 2011, with DOA between 310° and 320°, and the Kuril Trench, with DOA between 325° and 345° (Fig. 1a), are present in the data for the entire analysis period. The DOA between 310° and 315° are particularly associated with slow-slip activity offshore east of Honshu, Japan^47^. The DOA between 320° and 325° are associated with a main shock at 18:07:59 UTC on 05 September 2018 with a magnitude of 6.7 at Hokkaido, northern Japan, and its numerous aftershocks (Fig. 1a).

Discrete signal trains are identified in the true back-azimuth to Ioto starting on 03 September 2018 (Supplementary Fig. 3). The present hydroacoustic analysis suggests that the initial volcanic activity at Ioto ceased on 05 September 2018, restarted on 07 September, and then lasted until 09 September 2018. Hydroacoustic signals associated with the undersea eruptions captured by the local flyover observation on 12 September 2018 were however not detected at Wake Island. A signal train is identified a few degrees off the H11N back-azimuth to Ioto, on 15 September 2018 (Fig. 3 and Supplementary Fig. 3). This is attributed to the seismic activity taking place offshore Okinawa Island, southwestern Japan (Fig. 1a). The equivalent signal train can also be identified in the H11S result.

Overall the RMS amplitudes of the 6 to 60 Hz band-pass filtered raw data of both H11N and H11S increased by 10 dB, sometimes by up to a few tens of dB, above the usual ambient noise level during the entire month of September 2018 (Fig. 3), due to general increased seismic activity in Japan and across the Pacific. Despite this increased background noise level, the Ioto volcanic activity could be distinguished from other sources of underwater sounds by the present procedure.

Histograms of arrivals detected at H11N and H11S with the cross-correlation processing described above, for azimuths between 255° and 345° in increments of 1°, are shown in Fig. 3b,d. The total number of arrivals detected by this processing at H11N and H11S during the month of September 2018 are 7,239 and 8,342, respectively. The back-azimuths above 310° corresponds to arrivals from the Japan Trench and the Kuril Trench. It should be noted that with the 20-s cross-correlation time-window used to detect the arrivals from undersea volcanic eruptions, the number of earthquakes arrivals will be to some extent over-estimated, as signals from earthquakes generally last longer than 20-s. A more detailed sensitivity analysis of the length of time-window versus number of detected events was not conducted in this study.

Of particular note is the fact that H11N was apparently less sensitive in detecting the hydroacoustic arrivals originating from the Ioto volcanic activity compared to H11S, even though they appear to have equal sensitivity in detecting the seismic activities which followed the Hokkaido earthquake. The explanation proposed for this difference is the hydroacoustic propagation path between Ioto and H11N, where lateral (out-of-plane) diffraction caused by shallow bathymetric features located on some of the geodesic propagation paths between Ioto and H11N may affect the direction of arrival of the signals, or may even shadow some of the arrivals. Further details are discussed in Supplementary Fig. 4.

Another difference in the histograms is that the H11S histogram shows a peak at a back-azimuth of 273°. This peak is attributed to the occurrence of a ship-based seismic survey based on the characteristics of the signals^48^. These survey signals were recorded within a period of one hour only on 20 September 2018 (Supplementary Fig. 5).

## Discussion

### Previous relevant observations from an undersea eruption at nearby Fukutoku-Okanoba in 2010

One of the objectives of the present study is the discrimination between volcanic earthquakes and undersea eruptions in the hydroacoustic data. The limited availability of ground truth observations limits the ability to use hydroacoustic signals from remote recordings to achieve this goal. However, one such dataset with ground truth visual observations which can be associated with a signal recorded at the HA11 remote hydrophones is available from Fukutoku-Okanoba. Fukutoku-Okanoba is a submarine volcano (rising to 29 m below the sea-surface) located 55 km south of Ioto (Fig. 1b). The volcano exhibited a number of eruptions on 03 February 2010^49^ (Supplementary Fig. 6a). These eruptions have been previously documented with a time-stamped visual aerial observation^50^. According to the JMA Monthly Report, a flyover at 05:27 UTC on 03 February 2010 showed a steam-and-ash plume rising to an altitude of approximately 100 m, accompanied by a yellow-green discoloration of the surrounding sea water (Supplementary Fig. 6b).

Waveforms associated with the 05:27 UTC explosive eruption were recorded at HA11 about 30 minutes later. Supplementary Fig. 6c shows the waveform for this event, the RMS amplitude, and the spectrogram obtained by the same processing used to generate Fig. 2a. The 10-s duration signal recorded by H11S1 at 05:56:50 UTC is associated with the Fuktoku-Okanoba 05:27 UTC undersea eruption based on the travel time from Fukutoku-Okanoba and the direction of arrival. In the spectrogram (Supplementary Fig. 6c), the signal can be distinguished from the ambient noise in the frequency band up to and above 60 Hz. The low frequency arrivals at 05:57:30 UTC and beyond were associated with an earthquake of magnitude 3.7 which occurred in Hokkaido, northern Japan, at 05:19 UTC. The high frequency arrival with 10-s duration at 05:58:00 UTC was not associated with the Fukutoku-Okanoba activity based on the direction of arrival (335.3°) and a large cross-correlation residual.

For further comparison, PSDs, derived using a 20-s time-window of the signal received at H11S1 from the Fukutoku-Okanoba undersea eruption and ambient noise are compared in Supplementary Fig. 6d. The PSD associated with the recorded Fukutoku-Okanoba undersea eruption is approximately 10 dB higher than the ambient noise in the frequency range between 20 Hz and 40 Hz. Although the active period of the Fukutoku-Okanoba undersea eruption in 2010 was limited in time, and no direct observation other than the visual sighting is available, this episodic event provides some substantiation of the method used in this paper to characterize the undersea eruptions which appear to have occurred in the vicinity of Ioto.

### Characteristics of hydroacoustic signals associated with explosive undersea eruptions remotely acquired at HA11

IMS HA data containing signals from undersea eruptions have been examined in previous hydroacoustic studies. Hydroacoustic signals recorded by HA11 originating from the undersea eruption of South Sarigan seamount in the Northern Mariana Islands in 2010 have been previously presented^44,51^. On-land seismic recordings taken approximately 10 to 30 km away from the source during the undersea eruption of South Sarigan seamount in 2010, have made it possible to study the detailed sequence of seismic activity^52^. This seismic study conducted nearby the volcano could identify the main plume-producing explosions with an accurate time stamp. These explosions started around 11:48 UTC on 29 May 2010. The duration of the explosive undersea eruptions was approximately 10 min. An example of the hydroacoustic signals corresponding to the explosion series received at H11S1 is shown in Supplementary Fig. 7a. The explosion series continued for up to 10 minutes and generated hydroacoustic signals comprised of clusters of discrete short-impulsive (less than 10-s) events with broadband frequency content.

More recently, HA11 data associated with undersea eruptions and precursory events from Ahyi seamount in the Northern Mariana Islands in 2014 have been examined^9,53^. The seismic signals detected by the regional seismic network could be linked to the far-field hydroacoustic signals received by HA11^9^. The majority of the Ahyi seamount undersea eruptions was characterized by short impulsive events starting at 00:05 UTC on 24 April 2014 and lasted 2 weeks. An example of H11S1 hydroacoustic signals for this period, presented in the same form as Supplementary Fig. 7a, is shown in Supplementary Fig. 7b. Each impulsive event is up to 10-s duration and covers a broad frequency band. The appearance of each impulsive event is similar over time, in which a large-amplitude event was exhibited followed by a small-amplitude one arriving with a delay of 20-s. We interpret that such a pattern of delayed arrivals may be consistent with a multi-path associated with reflections from bathymetric features. However, the investigation of this particular aspect is beyond the scope of this paper.

After a few hours, the initial pattern of the Ahyi seamount undersea eruptions changed slightly, with arrivals occasionally occurring in clusters of several impulsive events within a time window of approximately one minute^9^.

### Interpretation of the Ioto volcanic activity using hydroacoustic signals acquired at HA11

As evidenced by the Fukutoku-Okanoba submarine eruption captured by the remote hydrophones and by the South Sarigan and Ahyi eruptions discussed above, a hydroacoustic signal associated with an undersea eruption can be characterized by a relatively short duration compared to signals originated from seismic sources. Furthermore, the examples of the explosive events at South Sarigan seamount and Ahyi seamount show that hydroacoustic signals associated with undersea eruptions can consist of clusters of discrete, short and impulsive signals with broadband frequency content. Therefore, we interpret that the short duration signals with high frequency content originating from Ioto volcano were caused by undersea eruptions, because they resemble explosive signals from South Sarigan and Ahyi. The hydroacoustic recordings at HA11 were analyzed also with respect to their frequency content, in order to further aid in the interpretation of the signals.

To achieve this, two band-pass filters were introduced prior to cross-correlation processing: (I) the broad pass-band between 6 Hz and 60 Hz discussed in the Results section above, and (II) a narrower high-frequency pass-band between 20 Hz and 60 Hz. While signals processed by (I) might have originated from either an undersea eruption or a seismic signal, ones processed by (II) are more readily interpreted as originating from an undersea eruption, based on the characteristics of previous known events recorded at HA11.

Whilst applying the high-frequency band pass filter (II) is expected to enable the detection of events which are interpreted as volcanic eruptions based on their high frequency content, it should be noted that in cases where the signal-to-noise ratio is low in band (II) the cross-correlation processing may provide a wrong estimate of the direction of arrival. For example, the signal at 21:34:24 UTC shown in Fig. 2a has a direction of arrival consistent with Ioto when using the frequency band between 20 Hz and 60 Hz before cross-correlation processing, while the one at 21:35:44 UTC yields a direction not consistent with Ioto. To circumvent this problem, we use changes in the RMS amplitude for the detection of short duration signals in the frequency band between 20 Hz and 60 Hz, while using the full band (I) for the determination of the back-azimuth of those arrivals.

To accomplish this, we implement the processing steps below:

- The RMS amplitude gradient of the time-series filtered in the frequency band between 20 Hz and 60 Hz is calculated using a 15-s bin, because the cross-correlation processing provides back-azimuth with a 15-s resolution (resulting from a 20-s time-window with 5-s overlap).
- If the RMS amplitude gradient at a certain time is larger than 6 dB re 1 count per second between neighboring bins, and the time is consistent with the direction of arrival based on the cross-correlation analysis using the full frequency band between 6 Hz and 60 Hz to compute the back-azimuth, then such a signal is counted as a short duration arrival coming from an undersea eruption of Ioto.

**Figure 4:**
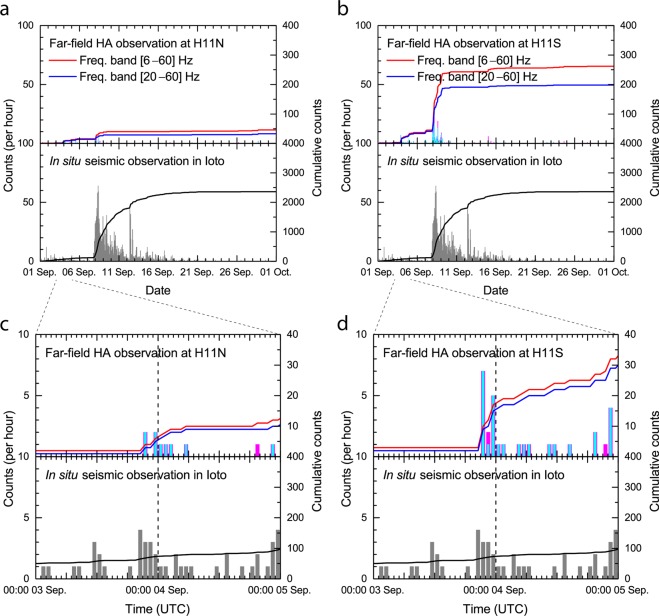
Comparison of HA11 hydroacoustic arrivals associated with Ioto (magenta, red and cyan, blue, representing frequency pass-bands I and II respectively) and the *in-situ* seismic measurements (grey). The data of the *in-situ* observation on Ioto was provided by the JMA. (**a**) The curve in the top panel shows the cumulative detections of H11N hydroacoustic signals, the curve in the bottom panel shows the equivalent cumulative detections for the Ioto *in-situ* seismic signals. The threshold for the detection of the *in-situ* seismic signal is displacement velocity higher than 30 μm/s and an S-P time of less than 2-s. Histograms show counts per hour. (**b**) Cumulative and hourly detections at H11S in the same form as (**a**). (**c**,**d**) Focus on the initial stage of the Ioto volcanic activity during two days from 03 to 05 September 2018.

Counts of arrivals within ±1° of the true back-azimuth to Ioto obtained by this processing from the time series recorded at the HA11 North and South triplets during September 2018 are presented in the histograms of Figs. 4a and 4b, respectively. The magenta and cyan bars are the number of detections per hour in the corresponding frequency pass-bands I and II, and the red and blue lines are cumulative detections since 01 September 2018. *In-situ* detections of volcanic seismicity performed by the JMA are also shown in grey, for a seismometer displacement velocity detection threshold higher than 30 μm/s and an S-P time of less than 2-s. Each bin represents the number of detections per hour, and the black lines are cumulative detections since 01 September 2018.

In the *in-situ* seismic data, although occasional signals were identified from the beginning of September, a marked increase can be observed at the end of 07 September 2018 (Fig. 4). The peak of volcanic seismicity recorded by the *in-situ* seismometers occurred at 08:00 UTC on 08 September 2018, with 64 detections per hour and 772 detections per day. This activity then gradually decreased until 12 September 2018. On 12 September 2018 when a small splash was confirmed by the local flyover observation, the volcanic seismicity spiked again for 4 hours, with up to 44 detections per hour. After 12 September 2018 the volcanic seismicity gradually decreased until 21 September 2018. During the month of September 2018 a total of 2,360 detections were identified as volcanic seismicity by the *in-situ* seismometers at Ioto.

The comparison of histograms between the remote hydrophone recordings and the *in-situ* seismometer confirms that the signals at HA11 originated from the Ioto volcanic activity (Fig. 4). For both frequency pass-bands, H11N shows fewer detections than H11S, as mentioned above, although the general patterns are similar between the two triplets.

The far-field hydroacoustic detections at HA11 commenced at 21:00 s UTC on 03 September 2018 (Fig. 4c,d). The period between 21:00 UTC on 03 September 2018 and 02:00 UTC on the following day showed high activity with respect to hydroacoustic observation at HA11, during which a total of 16 detections are associated with the Ioto volcanic activity. Of these 16 detections 15 were interpreted to be undersea eruptions based on the frequency content and short duration criterion.

The total number of hydroacoustic detections from far-field recordings by H11S is comparable to the *in-situ* seismic observations at Ioto during this initial period. After this period, there are more detections from the *in-situ* seismic observations than from the far-field hydroacoustic observations. This may indicate that the early stage of the volcanic activity of Ioto (from 03 September 2018 to 04 September 2018) was more efficient at producing hydroacoustic signals compared to the later stages.

The analysis of the H11S data, using the two frequency pass-band criterion described above, identified 262 detections originating from Ioto during the entire month of September 2018, in which 198 detections were interpreted to be associated with undersea eruptions. The results based on the frequency content of the data recorded remotely by the HA11 station thus suggest that around 75% of the arrivals originating from Ioto during the month of September 2018 can be associated with undersea eruptions, although uncertainties remain due to the lack of ground truth. The total number of 262 detections derived within the full-frequency band, i.e. between 6 Hz and 60 Hz, represent just 11% of the overall *in-situ* seismic observations. During the initial stage of the Ioto volcanic activity on 03 September 2018, the agreement between the far-field hydroacoustic observations and the *in-situ* data was closer than during the later phase. This fact suggests that most of the later Ioto volcanic activity was too weak to generate hydroacoustic signals that could be detected at HA11. The authors could also not exclude the possibility that some of the seismicity detected by the *in-situ* seismometers on Ioto occurred on the far side of the island, i.e. in such a location that the island itself acted as a blockage for direct propagation to HA11, or that the earthquake sources were deep, thus affecting the ability to produce a hydroacoustic signal detectable at HA11.

The analysis of hydroacoustic data recorded at the IMS hydrophone station HA11 presented here shows that the remote hydrophones appear to be capable of detecting and identifying signals from submarine volcanic eruptions at distances of 1,000’s of kilometers. Noting that the IMS comprises a total of six hydrophone stations (HA01 Cape Leeuwin (Australia), HA03 Juan Fernandez Islands (Chile), HA04 Crozet Islands (French Southern and Antarctic Territories), HA08 Diego Garcia (British Indian Ocean Territories), HA10 Ascension Island (United Kingdom) and HA11 Wake Island (U.S. Territory), all of which transmit continuous raw data in real-time to the CTBT’s IDC in Vienna, Austria) this study highlights the potential usefulness of the IMS hydroacoustic stations for listening to the oceans in search of submarine volcanic activity, particularly in areas which are not accessible for local monitoring.

## Supplementary information

Supplementary information
