# Supplementary material for: Interpretation of detections of volcanic activity at Ioto Island obtained from in situ seismometers and remote hydrophones of the International Monitoring System
Source: Sci Rep. 2019 Dec 20;9:19519. doi: 10.1038/s41598-019-55918-w (PMC6925123; doi:10.1038/s41598-019-55918-w)
Supplement: Supplementary file 1 — Supplementary information [file 41598_2019_55918_MOESM1_ESM.pdf]

Interpretation of detections of volcanic activity at Ioto Island obtained from *in situ* seismometers and remote hydrophones of the International Monitoring System

Hiroyuki Matsumoto[1, 2], Mario Zampolli[2], Georgios Haralabus[2], Jerry Stanley[2],
James Mattila[2] & Nurcan Meral Özel[2]

[1] Japan Agency for Marine-Earth Science and Technology (JAMSTEC)
2-15, Natsushima, Yokosuka 237-0061, Japan

[2] Comprehensive Nuclear-Test-Ban Treaty Organization (CTBTO)
Vienna International Centre, P. O. Box 1200, 1400 Vienna, Austria

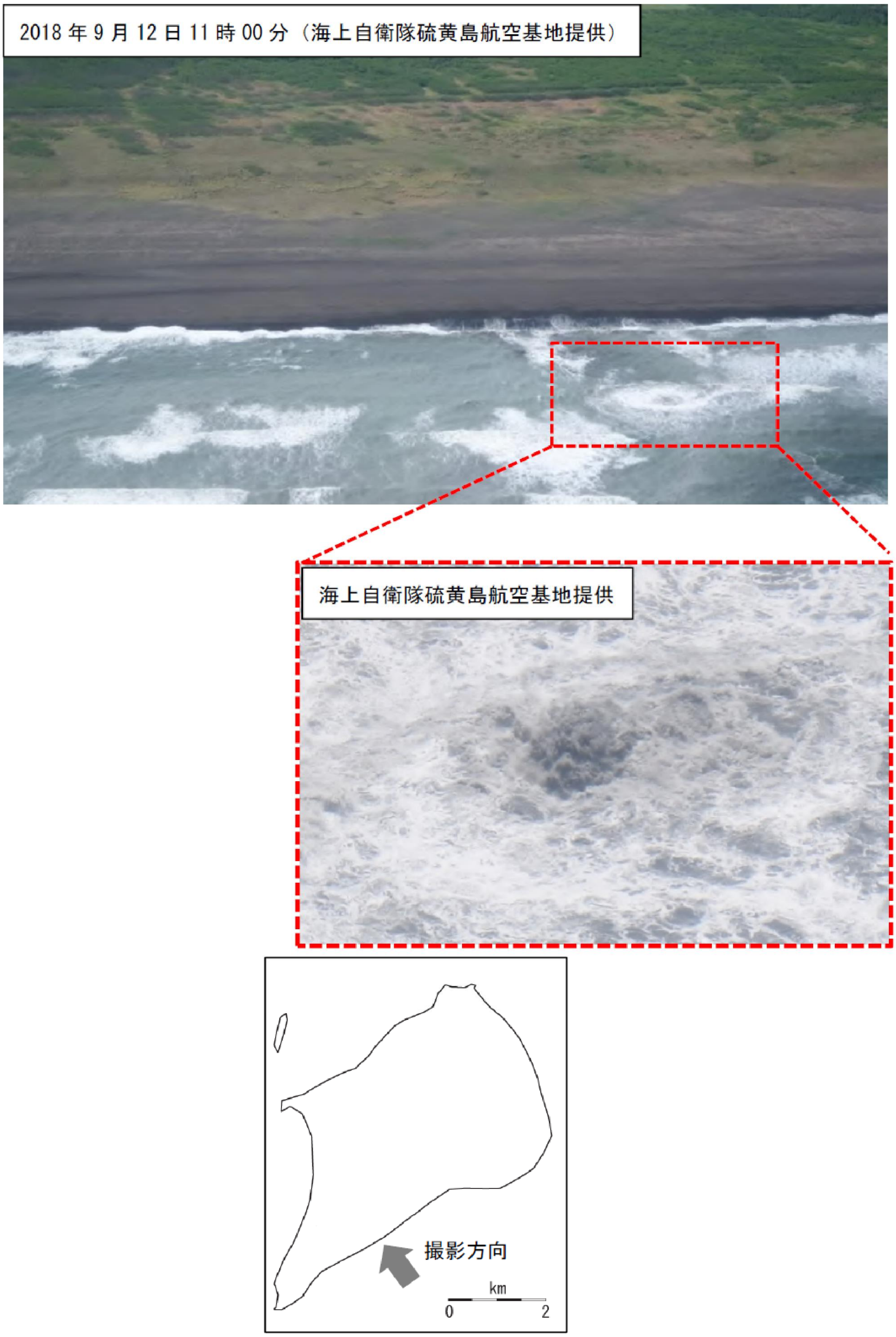

図１　硫黄島　硫黄島南側沿岸部の様子（2018 年９月 12 日、海上自衛隊硫黄島航空基地撮影）
・海水が海面から５～10mの高さまで噴出している様子。海底噴火が発生したと推定される。

Supplementary Figure 1: Local flyover photo near the shoreline of Ioto. This photo, which was the only one available to the public, was taken at 02:00 UTC (11:00 Local Time) on 12 September 2018. The source is the Monthly Report issued by the Japan Meteorological Agency (JMA) (https://www.data.jma.go.jp/svd/vois/data/tokyo/STOCK/monthly_v-act_doc/tokyo/18m09/329_18m09.pdf). Translation of the figure caption from Japanese: ñFigure 1 Ioto. Flyover photo of the south coast area of Ioto (on 12 September 2018, Photo taken by the Iwo-to Base of the Japan Maritime Self-Defense Force (JMSDF)) Sea water splashes as high as 5 to 10 m high. Undersea eruptions possibly occurred.ò

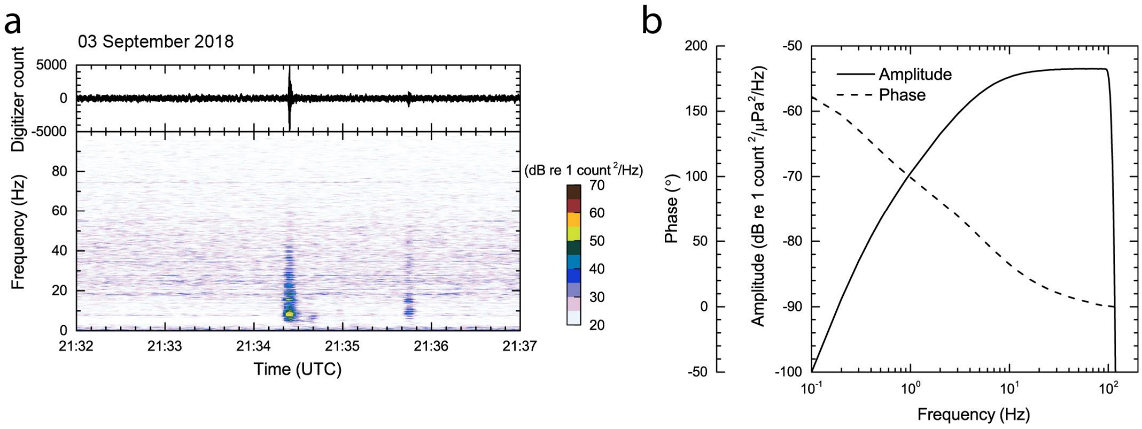

Supplementary Figure 2: (a) H11S1 hydrophone raw data and spectrogram during the Ioto undersea eruptions on 03 September 2018. The period presented is the same as Article Figure 2(a). Figure (b) shows the Frequency-amplitude-phase (FAP) response of the HA11 hydrophones. This FAP response was used for the sensorôs response correction shown in Figure 2.

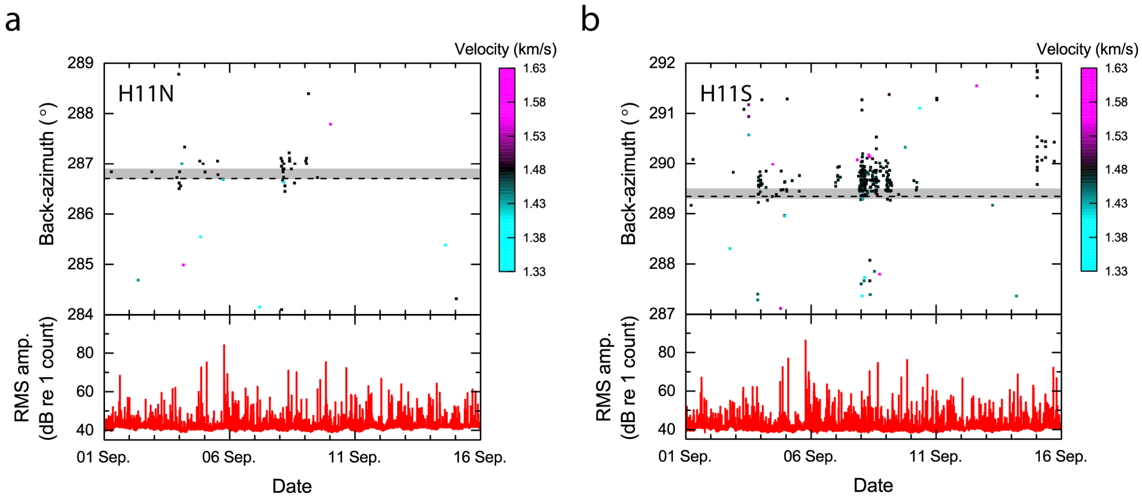

Supplementary Figure 3: Cross-correlation analysis of data focusing on back-azimuth around Ioto during the period between 01 and 16 September 2018 for (a) North and (b) South triplets. Template presented is the same as Article Figure 3 (a) and (c). The shaded back-azimuth zone corresponds to the direct geodesic rays spanning the extent of Ioto which is exposed above the sea-surface.

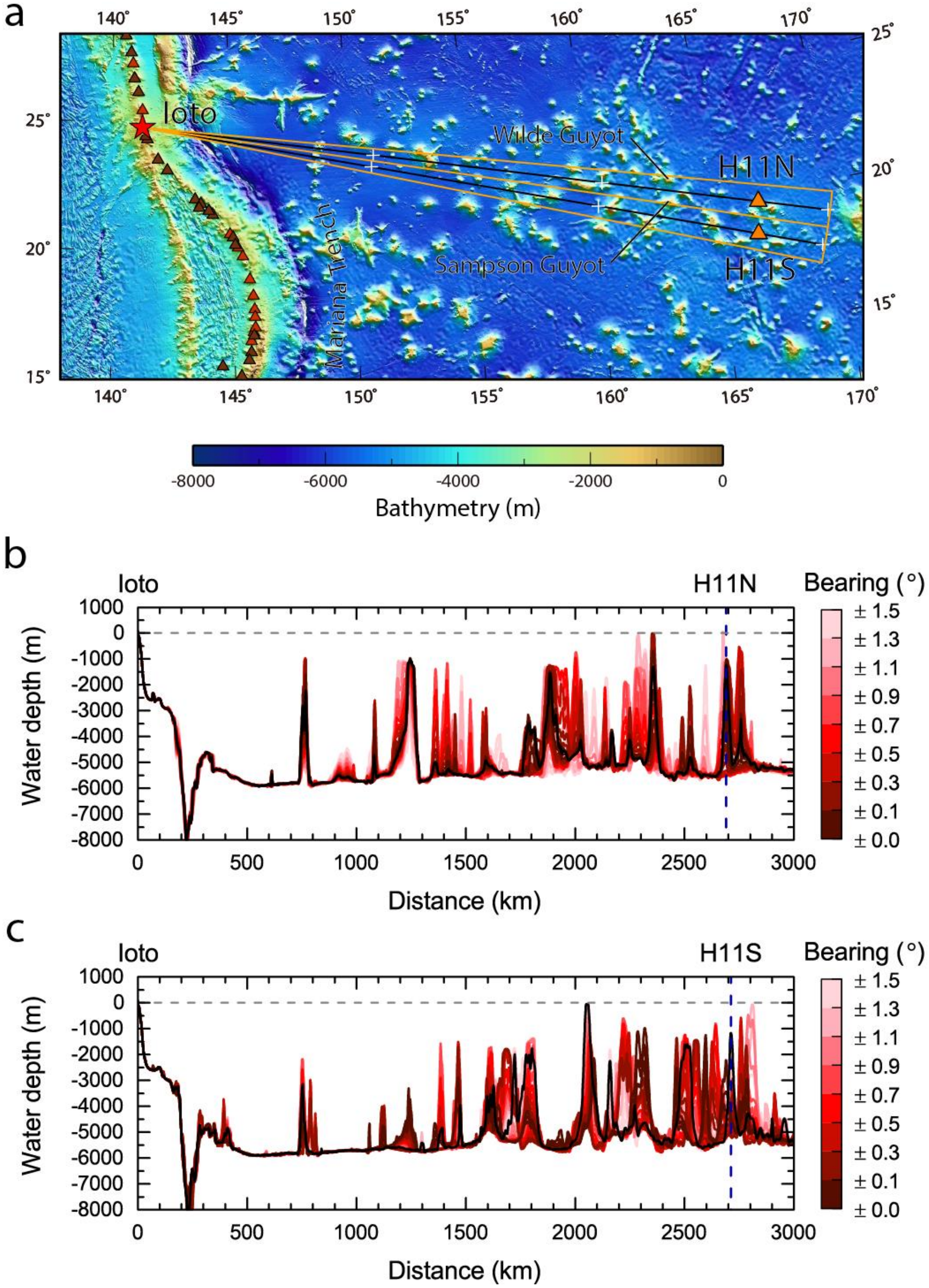
a
Ioto
Wilde Guyot
H11N
H11S
Sampson Guyot
Mariana Trench
Bathymetry (m)
-8000
-6000
-4000
-2000
0
b
Ioto
H11N
Bearing (°)
Water depth (m)
Distance (km)
± 1.5
± 1.3
± 1.1
± 0.9
± 0.7
± 0.5
± 0.3
± 0.1
± 0.0
c
Ioto
H11S
Bearing (°)
Water depth (m)
Distance (km)

Supplementary Figure 4: Bathymetric profiles between Ioto and HA11 derived using the GEBCO 30-arc-seconds resolution dataset55. (a) Black lines show the geodesic direct paths between Ioto and H11N and H11S using the WGS-84 datum. White crosses on the lines indicate distance increments of 1000 km from Ioto. The orange wedges detail an area of ± 1.5° around the central direct path. Figures (b) and (c) are the cross-sectional profiles from Ioto to H11N and H11S, respectively. The black line represents the bathymetry along the direct path, the color coding showing the angle w.r.t. the direct path along which the bathymetry is plotted. The figures show that an atoll is located along the direct path from Ioto to the South triplet, at a distance of approximately 600 km from H11S. This atoll appears to not interfere with the paths that are near the direct path (out-of-plane path), as shown by the cross-sectional bathymetry profiles for angles other than 0° in the figure. The paths which are within ± 0.1° from the direct path reveal that there is a shallow sea-mount at less than 400 km from H11N. It is possible that such a bathymetric feature, closer to the HA11 triplet, could affect the detection of signals from Ioto at the North triplet because of lateral diffraction, preventing the detection of those arrivals which had a lower source level at the origin.

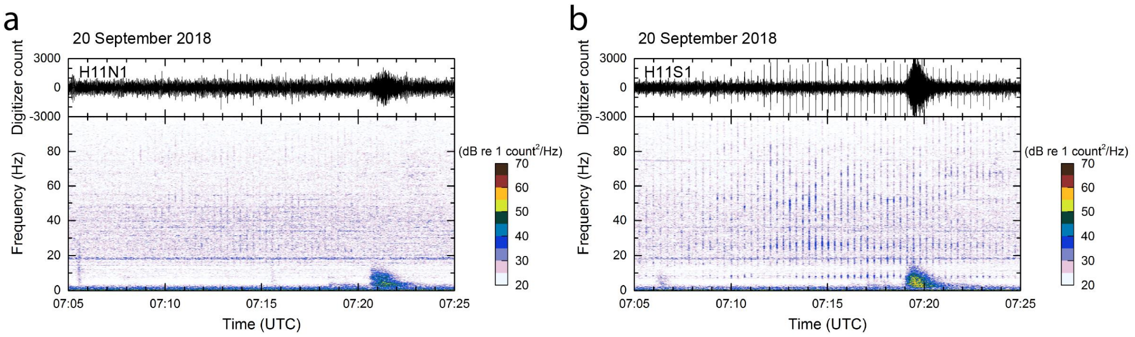

Supplementary Figure 5: Examples of hydrophone raw data and spectrograms of (a) H11N1 and (b) H11S1 recordings of air-gun shots on 20 September 2018. Such short and broadband signal features, with a regular repetition rate of 20-s, are typically associated with geophysical off-shore surveys.

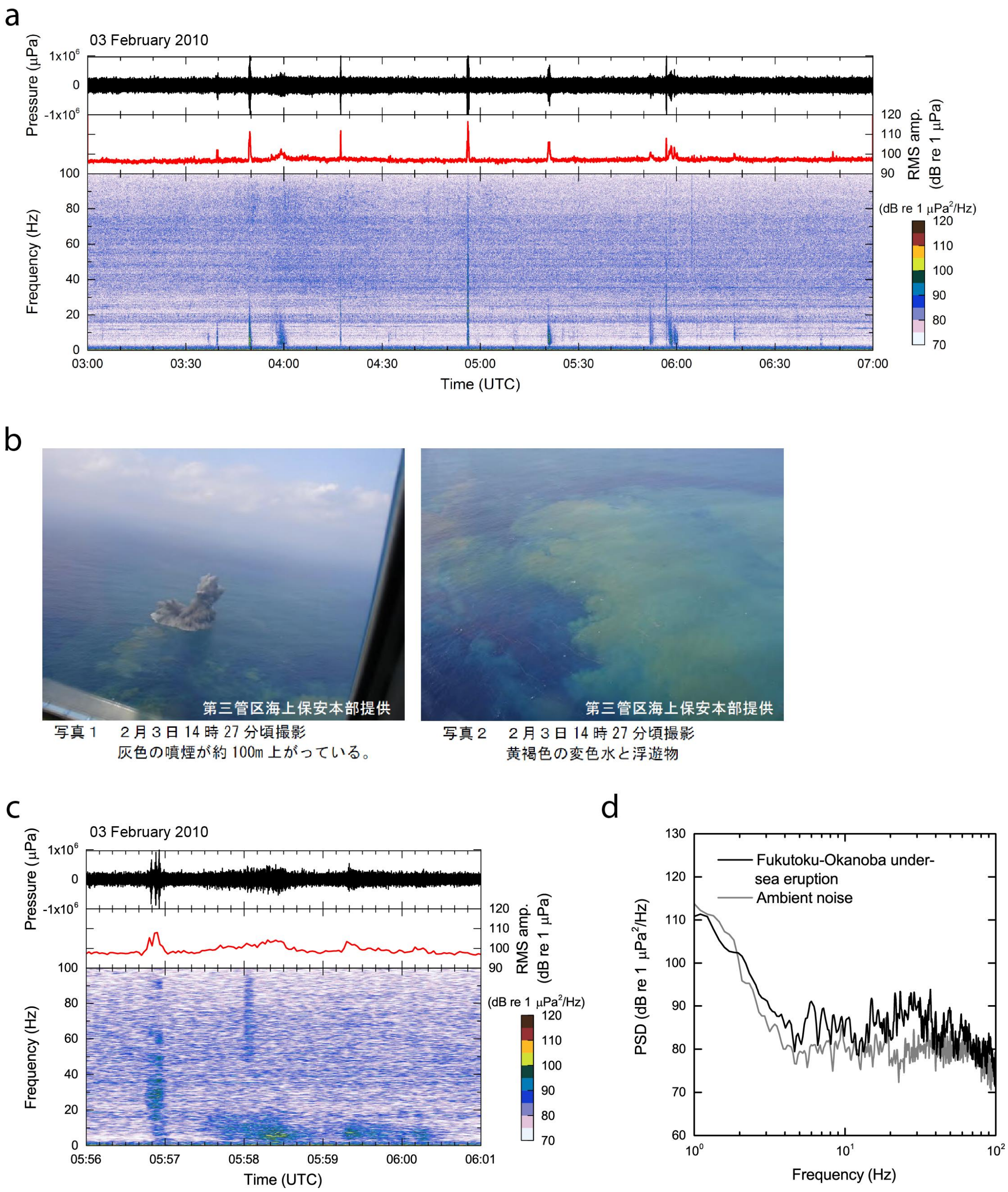

Supplementary Figure 6: Fukutoku-Okanoba undersea eruption on 03 February 2010. (a) H11S1 hydrophone data, RMS, and spectrogram for an arbitrary period of 4 hours during the day. (b) Photographs taken at 05:27 UTC on 03 February 2010 by the Japan Coast Guard (JCG). The source is the Monthly Report issued by the JMA (https://www.data.jma.go.jp/svd/vois/data/tokyo/STOCK/monthly_v-act_doc/tokyo/10m02/331_10m02.pdf). A jet of material rising up to 100 m above the sea-surface (left)

and discolored water (right) were observed. A flyover observation identified that an undersea eruption took place at 05:27 UTC on 03 February 2010. (c) Based on time of arrival and back-azimuth it was possible to associate the signal received at 05:56:50 UTC with the observed Fukutoku-Okanoba undersea eruption shown in Figure (b). The successive longer-lasting (> 2-min) low-frequency (< 20 Hz) signal is typical of an earthquake and is not associated with the eruption. The short duration (< 10-s) high-frequency (> 50 Hz) signal received at 05:58:00 UTC was not associated with the Fukutoku-Okanoba event because the direction of arrival (335.3°) was not compatible with a source at Fukutoku-Okanoba. (d) PSDs of the Fukutoku-Okanoba undersea eruption received over the time period of 05:56:40 UTC to 05:57:00 UTC and ambient noise.

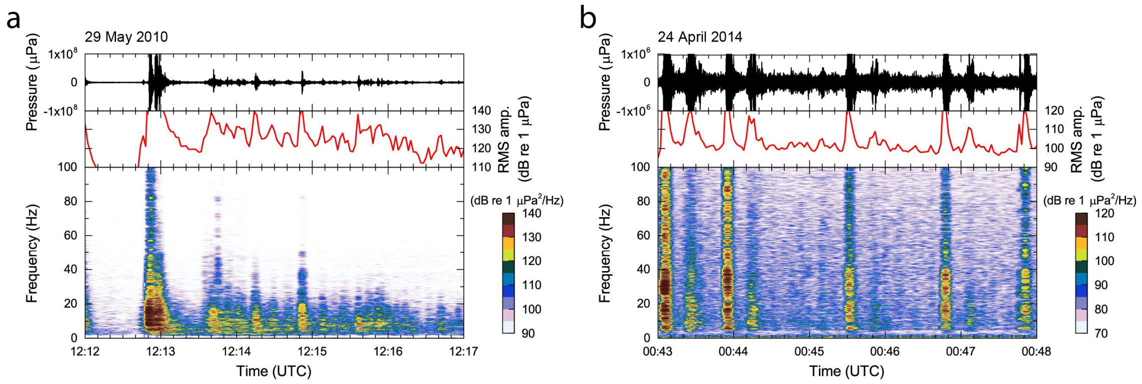

Supplementary Figure 7: Examples of H11S1 data associated with explosive undersea eruptions of (a) South Sarigan seamount in 2010 and (b) Ahyi seamount in 2014. Note that the color-scale used in the left panel is different from the one of the other plots shown in the article, although the processing that was used to generate the spectrograms is the same.
